# Increased Sensitivity to Mirror Symmetry in Autism

**DOI:** 10.1371/journal.pone.0019519

**Published:** 2011-04-29

**Authors:** Audrey Perreault, Rick Gurnsey, Michelle Dawson, Laurent Mottron, Armando Bertone

**Affiliations:** 1 Perceptual Neuroscience Lab for Autism and Development, Montreal, Quebec, Canada; 2 University of Montreal Center of Excellence for Pervasive Developmental Disorders (CETEDUM), Montreal, Quebec, Canada; 3 Department of Psychology, Université de Montréal, Montreal, Quebec, Canada; 4 Department of Psychology, Concordia University, Montreal, Quebec, Canada; 5 Department of Psychiatry, Université de Montréal, Montreal, Quebec, Canada; Istituto di Neuroscienze, Italy

## Abstract

Can autistic people see the forest for the trees? Ongoing uncertainty about the integrity and role of global processing in autism gives special importance to the question of how autistic individuals group local stimulus attributes into meaningful spatial patterns. We investigated visual grouping in autism by measuring sensitivity to mirror symmetry, a highly-salient perceptual image attribute preceding object recognition. Autistic and non-autistic individuals were asked to detect mirror symmetry oriented along vertical, oblique, and horizontal axes. Both groups performed best when the axis was vertical, but across all randomly-presented axis orientations, autistics were significantly more sensitive to symmetry than non-autistics. We suggest that under some circumstances, autistic individuals can take advantage of parallel access to local and global information. In other words, autistics may sometimes see the forest and the trees, and may therefore extract from noisy environments genuine regularities which elude non-autistic observers.

## Introduction

Autism is a neurodevelopmental variant whose current diagnostic criteria describe overt behavioral atypicalities in three domains: social interaction, communication, and restricted interests and repetitive behaviors [1]. Research addressing the interrelated social and communication domains has traditionally been dominant. In contrast, there has been relatively less impetus for understanding behaviors encompassing unusual, intense, and narrow interests or preoccupations (e.g., with specific aspects of objects or the environment), as well as repetitive routines or motor mannerisms. However, recognition that this putatively “non-social” domain is in fact important for identifying and understanding the autistic behavioral phenotype is increasing [2]. Several recent findings demonstrate that focused interests and repetitive behaviorsrelated to visual perception—such as unusual visual exploration (e.g., lateral glances), longer fixations, and frequent spinning of objects—are common in and specific to autism starting early in development [3]–[5].

The origin of such atypical autistic visual behaviors is as yet unknown. One proposal is that atypical development of perceptual functions ultimately results in a perceptual signature or profile that distinguishes autism from both typical development and other neurodevelopmental conditions [6], [7]. This signature takes into account findings of superior performance by autistics on a variety of visuospatial tasks, including visual search, block design, and embedded figures tasks [8]–[10]. There is also preliminary evidence that autistics have difficulty processing elementary visual attributes such as texture and color [11], [12].

Together with interest in the understudied “non-social” domain, interest in the neural underpinnings mediating early perceptualabilities in autism has recently proliferated [13], [14]. Studies have been interpreted largely within the context of two evolving neurocognitive models, whose tenets differ with regard to the origin of recurrent findings of enhanced autistic performance on several types of visual tasks. The first model, weak central coherence [15], posits that superior performanceon visuospatial tasks is the result of an apparent local processing bias when locally-oriented analysis is considered to be advantageous. According to WCC, the same local processing bias also predicts a defective construction of global visual representations, a perceptual trade-off analogous to not being able to see the forest for the trees. The second model, enhanced perceptual functioning [16], posits autistics' superior performance as reflecting an increased role and autonomy of perception during the completion of cognitive tasks. One possible mechanism is an increased functional involvement of early and associative perceptual cortices [17]. In this model, autistics are able to construct global representations but do so atypically, such that access to local information is not lost in favor of the efficient analysis of a global percept, as is the case within typical processing hierarchies.

The two models of autistic perception diverge with respect to the role of global processing and thus motivate an evaluation of grouping processes in autism. Understanding of elementary, local visual perception (e.g., motion, color, texture, etc.) is growing and beginning to complement the vast literature on socially-contingent object perception (e.g., face perception). Nevertheless, the question of how local stimulus attributes are grouped into meaningful spatial patterns has not yet adequately been studied in autism [18].The goal of this study was therefore to assess visual grouping in autism by measuring sensitivity to visual symmetry, a prototypical and ecologically-significant type of grouping that exemplifies how spatial information is organized before visual object perception occurs.

Mirror symmetry,where one half of a pattern is a mirrorreflection of the other half, is a highly salient visual attribute involved in figure-ground segregation and in object perception and recognition [19], [20]. The perception of mirror symmetry emerges from multiple stages of spatial processing. An encoding process, initiated by individual neurons or spatial filters, starts with the assimilation of local elements positioned at the same spatial location relative to, but on either side of, the symmetrical axis [19], [21]. These local-element pairs are subsequently integrated or grouped at a comparison stage before a globally symmetric patternis perceived [21]–[23]. The spatial filters compare information of similar contrasts present at two locations equidistant from a symmetry axis, andoutputs of pairs of detectors relative to a given symmetry axis are summed to form the symmetry signal relative to that location.

Although symmetry perception is initiated by local processing, the extraction of global symmetric configurations has been demonstrated to selectively solicit higher-order cortical visual brain areas, including V3A, V4, V7, and LO [24]. The importance of mirror symmetry with respect to object recognition is reflected by the fact that under conditions of uncertainty, symmetry is most efficiently perceived if oriented about a vertical axis [20], [23], [25], an advantage argued to reflect the ecological and social significance of most vertically symmetrical objects [20].

In the present study, we assessed the ability of autistics and matched non-autistics to detect mirror symmetry oriented about vertical, oblique, and horizontal axes. Adifferential ability to perceive symmetry would suggest different methods of global pattern extraction in autism, an essential level of perceptual analysis preceding object perception.

## Methods

### Participants

Seventeen autisticand 15 typically developing individuals, recruited from the Rivière-des-Prairies Hospital database, participated in the study. Autism was diagnosed using the Autism Diagnostic Interview – Revised (ADI-R)combined with the Autistic Diagnostic Observation Schedule - General (ADOS-G), both of which were conducted by a trained clinician-researcher (LM) who obtained reliability on these instruments.The comparison group was composed of non-autistic adolescents and adults screened with a questionnaire for personal or familial history of neurological or psychiatric disorders. Autistic and non-autistic participants were matched on gender, global IQ as measured by Wechsler Scales, and age (see Table 1). All participants had Wechsler scores of 80 or higher, and normal or corrected-to-normal far and near vision as assessed before testing using both near and far acuity charts (i.e. near point directional –E- and –C cards, Snellen letter sequence-A-new Logmar). The ethics committee at Rivière-des-Prairies Hospital approved the study. Participants or their parents (if under 18 years) provided written informed consent. The study was carried out in accordance with the Declaration of Helsinki, and was approved by the research ethics committee at l'HôpitalRivière-des-Prairies.

**Table 1:** Mean and Standard Deviations for Variables Used to Match Autistics to Non-Autistics.

	Autistic	Non-autistic	*t*&*p* values
Gender	14 males	15 males	
Chronological Age			*t*(27) = 1.83, *p* = 0.08
*M*	24.06	20.47	
*SD*	6.30	4.50	
Range	14–35	15–29	
Full Scale IQ			*t*(27) = −0.60, *p* = 0.56
*M*	102.88	105.40	
*SD*	12.90	10.66	
Range	81–126	88–122	
Performance IQ			*t*(27) = 1.18, *p = *0.25
*M*	109.42	104.93	
*SD*	8.31	10.79	
Range	96–121	87–119	
Verbal IQ			*t*(27) = −0.50, *p* = 0.62
*M*	103.50	105.27	
*SD*	16.07	12.83	
Range	77–128	91–127	

### Apparatus and stimuli

Stimulus construction, presentation, and data recording were controlled by Matlab-driven routines from the Psychophysics and Video Toolbox. All stimuli were displayed on a gamma-corrected 19-inch Viewsonic monitor with a screen resolution of 1152×870 pixels using a MACPRO G4 testing station. The mean luminance of the display was 20.00 cd/m^2^ (x = 0.2783, y = 0.3210 in CIE (Commission Internationale de l'Eclairage) u' v' color space). A Minolta CS-100 Chroma Meter colorimeter was used for the calibration and luminance readings.

**Figure 1:**
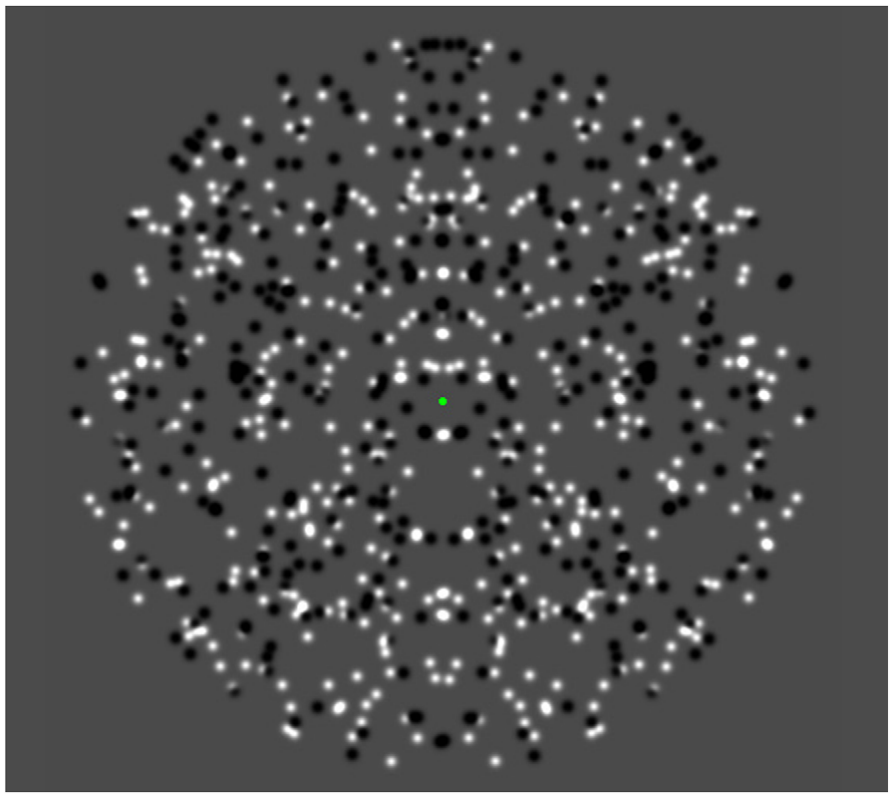
Example of typical symmetry pattern with a vertical axis of orientation. All stimuli were comprised of 500 dot pairs (half white and half black) presented within a circular aperture subtending 10° in diameter.

Symmetrical stimuli wereglobal patterns whose local elements, or dot-pairs, were located equidistant from either side of an axis [P_L_(x,y) and P_R_(-x,y)]. As shown in Figure 1, symmetrical dot pairs always shared the same luminance polarity, either black-black (1.0 cd/m^2^) or white-white (39.0 cd/m^2^). Symmetrical patterns (target stimuli) were composed of 500 dot-pairs (1000 total dots) presented within a circular aperture that subtended 10° in diameter when viewed at a distance of 57 centimeters; individual dots, comprising the dot-pairs, subtended ≈ 0.1 degrees at the same distance. Signal strength was determined by the proportion of dots matched across the axis of symmetry such that 0% matching meant the pattern was perfectly random, and 100% matching meant the pattern was perfectly symmetrical (as depicted in Figure 1). Based on pilot testing, seven levels of signal strength were chosen for experimentation(30%, 36.7%, 44.8%, 54.8%, 66.9%, 81.8% and 100% symmetrical dot-pairs). Symmetrical stimuli were presented withtheir axes orientated either vertically (0°), obliquely (45°), or horizontally (90°). Non-symmetrical (non-target) stimuli consisted of patterns where 0% of dot-pairs were symmetrical relative to the symmetry axis.

### Procedure

On each trialparticipants were asked to detect which of two successively-presented stimuli contained symmetry, with one stimulus containing no symmetry (non-target) and the other containing one of the seven predetermined symmetrical signal strength levels (target). Participants responded by pressing one of two keys on a keyboard. Each stimulus was presented for 250 ms, separated by a 100 ms grey screen. Within a testing block, each of the seven signal strengthswas presented at each of three orientations (vertical, horizontal, and oblique) in random order, resulting in 21 trials (3 orientations X 7 signal strengths). A complete testing session consisted of25 blocks, resulting in 25 measures for each of the 21 experimental conditions. A 5-minute practicesessioncontaining 5 trials of highly-visible symmetry patterns for each orientation preceded testing in order to familiarize participants with fixation, stimuli presentation, and responding. All experiments were conducted in a dimly-lit room. Throughout testing, participants were reminded to fixate the center of each pattern and were encouraged to take breaks if they felt tired or distracted. The experimenter remained present throughout testing to monitor fixation and fatigue. The entire testing session took approximately 60 minutes to complete.

## Results

Data for three of the 17 autistic participants were not used for analysis because the participants were unable to obtain threshold for any of the three symmetry conditions (i.e., vertical, oblique, or horizontal). Two of these participants were also unable to complete a separate orientation discrimination task, which suggests that their inability to reach threshold was due to difficulties in task comprehension rather than an inability to perceive symmetry.

**Figure 2:**
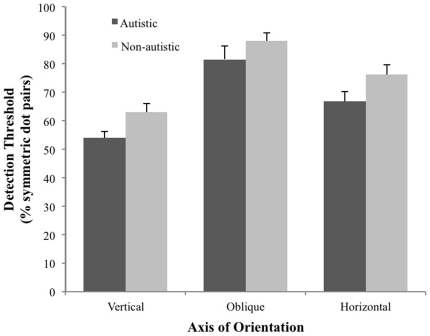
Mean symmetry detection threshold (+*SE*) for autistic (*n* = 14) and non-autistic (*n* = 15) groups plotted as a function of axis of orientation (vertical, horizontal, or oblique).

Symmetry detection thresholds were derived for each orientation by fitting a Cumulative Gaussian Distribution function to the signal strength vs. accuracy functions. Threshold was defined as the signal strength eliciting 75% correct responses.Figure 2 shows the mean symmetry detection thresholds as a function of axis of orientation for autistic (black bars) and non-autistic (gray bars) groups. As expected, a 2 (group) ×3 (orientation) mixed factorial design, with alpha level set at 0.05, revealed a significant main effect of axes of orientation (*F*(2, 54) = 37.78, *p*<0.05; *η^2^_partial_* =  0.58). Tukey post-hoc analysis, with alpha level set at 0.01, revealed that mean detection threshold for vertically-oriented symmetry (*M* = 58.85, *SD* = 10.66) was lower compared to both oblique (*M* = 85.48, *SD* = 14.53) and horizontal (*M* = 72.76, *SD* = 14.55) conditions.

A main group effect was also evident (*F*(1, 27) = 4.42, *p*<0.05; *η^2^_partial_* =  0.14), with mean symmetry detection thresholds significantly lower in the autistic group compared to the non-autistic group when averaged across axis of orientation. A group x orientation interaction was not found (*F*(2, 54) = 0.18, *p* = *ns*; *η^2^_partial_* = 0.01) as mean between-group differences in symmetry detection threshold did not significantly differ as a function of axis of orientation.

## Discussion

Our aim was to assess visual grouping in autism by measuring sensitivity to mirror visual symmetry, a salient attribute inherent in many visual objects. Our findings suggest that symmetry perception is both typical and atypical in autism. It is typical in that autistics were most sensitive to vertically-oriented symmetry patterns, an expected advantage also found in the non-autistic group. However, groups differed in overall sensitivity to visual symmetry across axes of orientation conditions, with autistics displaying enhanced performance compared to their non-autistic controls.

The typical “vertical advantage” for detecting mirror symmetry may reflect the ecological and/or social significance of vertically symmetrical objects, such as human faces, in our every-day environment. It has been proposed that autistics are innately insensitive to the importance of socially-relevant information, particularly human faces [26]. A failure of autistics to demonstrate the vertical advantage in visual symmetry detection would support this proposal. However, we found that autistics have the same vertical advantage as nonautistics.

At the same time, we found that visual symmetry perception was *atypical* in autism. Autistics were *more* sensitive to symmetry than their non-autistic controls across all three axes of orientation. By definition, the perception of visual symmetry is a grouping task that necessarily involves spatial integration. This finding is therefore inconsistent with the WCC-based hypothesis suggesting that while autistic spatial perception can be advantageous in the processing of local elements, it is defined by inefficient integrative analysis [15]. In a comparable contour integration task, Del Viva, Igliozzi, Tancredi, and Brizzolara (2006) found an equivalent ability of young autistics and non-autistics to detect the spatial position of a circularly-configured chain of local elements (signal) embedded in background noise [27]. Our findings, however, differ from those of previous relevant studies of grouping abilities in that autistics manifested a visuo-spatial processing *advantage*.

We propose that this superior performance may originate from autistics' efficacy at extractingrecurring complex regularities from noisy arrays of information. Symmetrical patternsare defined by multiple highly structured spatial relationships between local elements, always presented at equidistant locations relative to an axis. As suggested by Mottron, Dawson, and Soulières (2009), neural mechanisms involved in pattern detection may be particularly active in autism [28]. Further, atypically autonomous cognitive processes in autism may allow for the parallel, non-strategic integration of patterns across multiple levels and scales, resulting in autistics' ability to efficiently access and extract signal from noise at both local (i.e. symmetrical dot-pairs) and global (globalsymmetrical patterns) levels. In theory, such parallel access would be less likelyin non-autistic individuals, whose ability to use local information from early visual areas would be diminished due to typical globally-biased processing hierarchies. A parallel processing advantage would be especially pertinent in our study given the difficulty of the task. Specifically, the global orientation of each symmetrical pattern was presented randomly within testing blocks, making it more difficult to use global spatial relationships to efficiently detect symmetry [29].

In sum, we did not find evidence for the autistic visual grouping deficit predicted by WCC. Instead our findings raise the possibility that under some circumstances autistics are atypical in seeing both the forest and the trees, leading in this case to superior detection of mirror symmetry. Autistics' enhanced ability to detect genuine regularities within noisy stimuli deserves more attention [14], particularly as these complex abilities have been found in autistic toddlers [30]. In addition, recent findings have demonstrated an autistic preference for dynamic visual regularities at a young age [31]. Our findings suggest that while autistics are sensitive to stimuli attributes that are salient to non-autistics (i.e., vertical advantage), autistics may in addition detect and respond to environmental regularities which elude non-autistics.
